# Low‐dose aspirin in unexplained recurrent pregnancy loss: A systematic review and meta‐analysis

**DOI:** 10.1002/pmf2.70099

**Published:** 2025-11-22

**Authors:** Marianna Onori, Giuliana Beneduce, Matteo Figà, Fabio Sannino, Alessandro Petrecca, Tullio Ghi, Chiara Tersigni

**Affiliations:** ^1^ Facoltà di Medicina e Chirurgia Università Cattolica del Sacro Cuore Rome Italy; ^2^ UOC Ostetricia e Patologia Ostetrica, Dipartimento di Scienze della Salute della Donna, del Bambino e di Sanità Pubblica, Fondazione Policlinico Universitario A. Gemelli IRCCS Rome Italy

**Keywords:** acetylsalicylic acid, live birth rate, low‐dose aspirin, unexplained recurrent pregnancy loss

## Abstract

**Introduction:**

Recurrent pregnancy loss (RPL), defined as two or more consecutive pregnancy losses before 24 weeks of gestation, affects approximately 1%–2% of couples of reproductive ages. Despite comprehensive diagnostic work‐up, up to 40%–50% of cases remain unexplained (uRPL). Low‐dose aspirin (LDA) (50, 75, or 80 mg) administration during pregnancy has been proposed as a potential treatment to improve obstetric outcomes in women with uRPL. However, its efficacy in preventing pregnancy loss remains unclear. The aim of this systematic review and meta‐analysis was to evaluate the efficacy of LDA administration on the pregnancy outcomes of women with uRPL.

**Methods:**

We systematically searched for relevant studies in PubMed, Scopus, EMBASE, Google Scholar, and the Cochrane Library without language restriction from inception until April 2025. Randomized controlled trials (RCTs) that summarize the evidence regarding the effect of LDA on obstetric outcomes in women with uRPL were included. The counted data were analyzed using relative risk (RR) as the effect‐size statistic, and each effect size was assigned its 95% confidence interval (CI). This study was conducted and reported according to the Preferred Reporting Items for Systematic Reviews and Meta‐Analyses (PRISMA) guidelines. The primary outcome was the live birth rate (LBR), and secondary outcomes included preeclampsia and fetal growth restriction rates.

**Results:**

Three RCTs were included, involving a total of 656 participants. No significant improvements were observed in terms of LBR in uRPL treated with daily administration of a 50–80 mg aspirin compared to untreated uRPL women (RR = 0.97, 95% CI, 0.90–1.04, *p* = 0.37).

**Conclusions:**

Our findings suggested that a 50–80 mg daily dose of aspirin does not improve the LBR of women with uRPL. Further studies, specifically RCTs using a 150 mg dose of aspirin in uRPL, are needed to clarify the efficacy of acetylsalicylic acid in preventing placental insufficiency‐related pregnancy loss.

**PROSPERO Registration:**

CRD420251024932

## 1 INTRODUCTION

Recurrent pregnancy loss (RPL) is defined as two or more consecutive pregnancy losses before 24 weeks of gestation and affects up to 1%–2% of reproductive‐age couples [1, 2]. RPL is a major obstetric syndrome with a complex and multifactorial pathogenesis. Recognized etiologic factors include parental chromosomal and genetic abnormalities, antiphospholipid syndrome (APS), endocrine disorders, autoimmune diseases, and uterine anatomical abnormalities. However, causal or risk factors can be identified only in a half of cases following a comprehensive work‐up, and approximately 40%–50% of RPL remains unexplained (uRPL). In patients with APS, whose obstetrical history is usually complicated by placental‐related disorders like RPL, fetal growth restriction (FGR), and preeclampsia (PE), the preventative treatment relies on the combination of prophylactic dose of low molecular weight heparin (LMWH) and low‐dose aspirin (LDA) with a live birth rate (LBR) of 70%–80% [3]. In addition, the efficacy of LDA in preventing early PE onset, administered in pregnancy at a dose of 150 mg/day from 11–14 to 36 weeks of gestation, has been largely documented [4]. In particular, the proposed beneficial effects of aspirin in improving placental and endothelial function in pregnancy do not rely only on its antithrombotic mechanisms. Indeed, aspirin has been also shown to (a) reduce the release of soluble forms‐like tyrosine kinase 1 (sFLT‐1) from trophoblast cells and induce the production of vascular endothelial growth factor (VEGF), promoting angiogenesis [5]; (b) modulate cytokine production, reduce apoptosis, and interfere with cell aggregation and fusion, improving defective trophoblast implantation [6]; improve extra villous trophoblast (EVT) migration and invasion into the maternal uterine spiral arteries [7]. Intriguingly, Burton and Jauniaux have proposed that insufficient placental development is the common ground of a large spectrum of placental‐related obstetric disorders, namely miscarriage, PE, and FGR. In particular, when the defective invasion of the spiral arteries is the most severe, premature onset of placental blood flow and syncytiotrophoblast oxidative stress occur, leading to miscarriage. In case of less severe defective placentation, early‐onset form of PE associated with FGR would occur, while late onset PE would be the clinical manifestation of the mildest defective EVT invasion [8]. Consistent to this theory, women with a history of RPL display a higher prevalence of placental dysfunctional disorders, that is, fetal growth restriction, PE, stillbirth, and placental abruption, compared to the general obstetric population [9].

Based on this evidence, LDA administration in pregnancy might represent a valuable treatment option in the context of uRPL. However, the efficacy of LDA therapy in uRPL remains unclear and a subject of debate, since the results of available studies are often contrasting and influenced by the different dosages used and/or the heterogeneity of the population included [10]. The objective of this systematic review and meta‐analysis was to summarize the available evidence on the effect of LDA administration in pregnancy in uRPL women in terms of LBR.

## 2 METHODS

A systematic review and meta‐analysis was conducted to evaluate the effect of LDA on the LBR in women with uRPL. This study was performed and reported according to the Preferred Reporting Items for Systematic Reviews and Meta‐Analyses (PRISMA) guidelines [11]. The PRISMA checklist is shown in Table S1.

### 2.1 Selection criteria

All randomized controlled trials (RCTs) reporting the effect of LDA administration on LBR in women with uRPL, compared to women with uRPL receiving placebo only, were included in this study. uRPL was defined as two or more consecutive pregnancy losses before 24 weeks of gestation in the absence of any other recognized causes of RPL (uterine structural abnormalities, thyroid dysfunction, and antiphospholipid antibodies). Studies were excluded in case of (a) inclusion of RPL women with known etiology, (b) use of combined therapies, and (c) the absence of the placebo group.

### 2.2 Information sources, search strategy, and study selection

The full search strategy, including both free‐text terms and MeSH terms, is provided in the Supporting Information.

### 2.3 Data extraction

Two investigators (F.S. and A.P.) extracted independently the following data from included studies, by using a standardized data extraction form: first author; year; country; study design; definition of uRPL; RPL diagnostic work‐up; sample; population age; therapy timing; total number of experimental group (uRPL treated with LDA); live birth‐positive cases; live birth‐negative cases; PE‐positive cases; PE‐negative cases; FGR‐positive cases; FGR‐negative cases; total number of placebo group (untreated uRPL); live birth‐positive controls; live birth‐negative controls; PE‐positive controls; PE‐negative controls; FGR‐positive controls; FGR‐negative controls.

### 2.4 Risk of bias assessment

**FIGURE 1:**
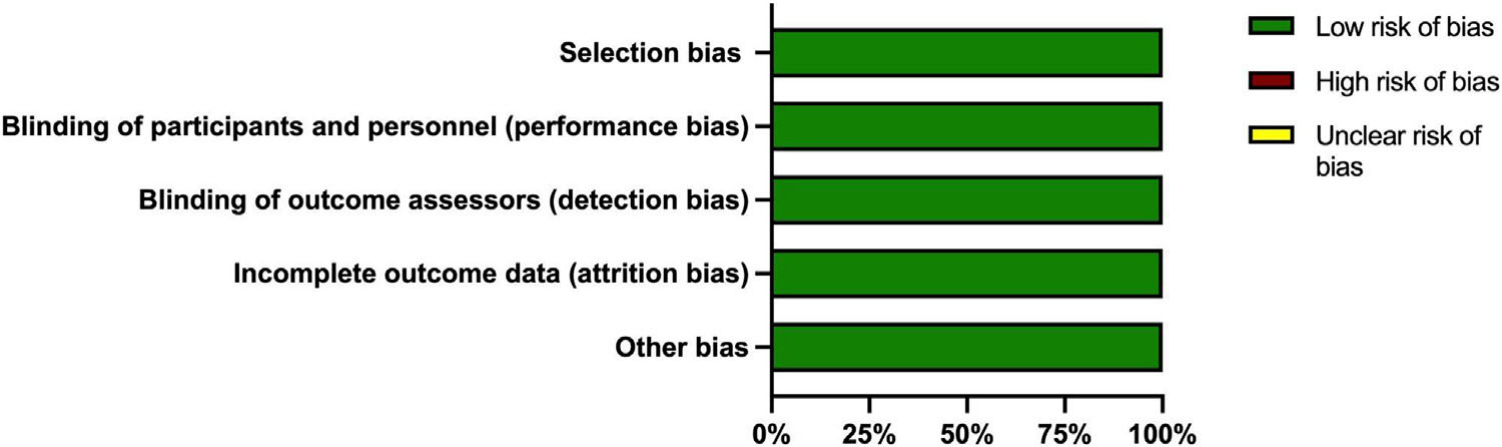
Risk of bias assessment of randomized controlled trials (RCTs) included in the meta‐analysis.

The JBI critical appraisal tool used to assess the methodological quality of studies included in meta‐analysis is shown in Figure 1 and Table S2. Questions are scored as ‘Yes’, ‘No’ and ‘Uncertain’.

The questions are as follows: ‘Q1. Was true randomization used for assignment of participants to treatment groups? Q2. Was allocation to treatment groups concealed? Q3. Were treatment groups similar at the baseline? Q4. Were participants blind to treatment assignment? Q5. Were those delivering treatment blind to treatment assignment? Q6. Were outcomes assessors blind to treatment assignment? Q7. Were treatment groups treated identically other than the intervention of interest? Q8. Was follow up complete and if not, were differences between groups in terms of their follow up adequately described and analyzed? Q9. Were participants analyzed in the groups to which they were randomized? Q10. Were outcomes measured in the same way for treatment groups? Q11. Were outcomes measured in a reliable way? Q12. Was appropriate statistical analysis used? Q13. Was the trial design appropriate, and any deviations from the standard RCT design (individual randomization, parallel groups) accounted for in the conduct and analysis of the trial?’.

Disagreements between reviewers were resolved by consensus or by the decision of a third independent reviewer (M.O.). Publication bias was not tested in this study because <10 studies were included in the analysis.

### 2.5 Synthesis methods

The pooled meta‐estimate was expressed as a risk ratio (RR) with 95% confidence intervals and visualized as a forest plot. Heterogeneity analysis of the studies was assessed using the standard Chi‐square test for heterogeneity (χ^2^) and *I* ^2^ statistics [12]. When the statistical heterogeneity among the studies was small (*I* ^2^ ≤ 50%), a fixed‐effect model was used; conversely, when the statistical heterogeneity among the studies was large (*I* ^2 ^> 50%), a random‐effect model was used. The JBI critical appraisal tools were used to assess the methodological quality of studies included [13]. Statistical analysis was performed using MetaAnalysisOnline [14]. For all analysis, *p* < 0.05 was considered significant.

### 2.6 Registration

This review was registered with the International Prospective Review of Systematic Reviews (PROSPERO) and the registration number is CRD420251024932.

## 3 RESULTS

### 3.1 Study selection

**FIGURE 2:**
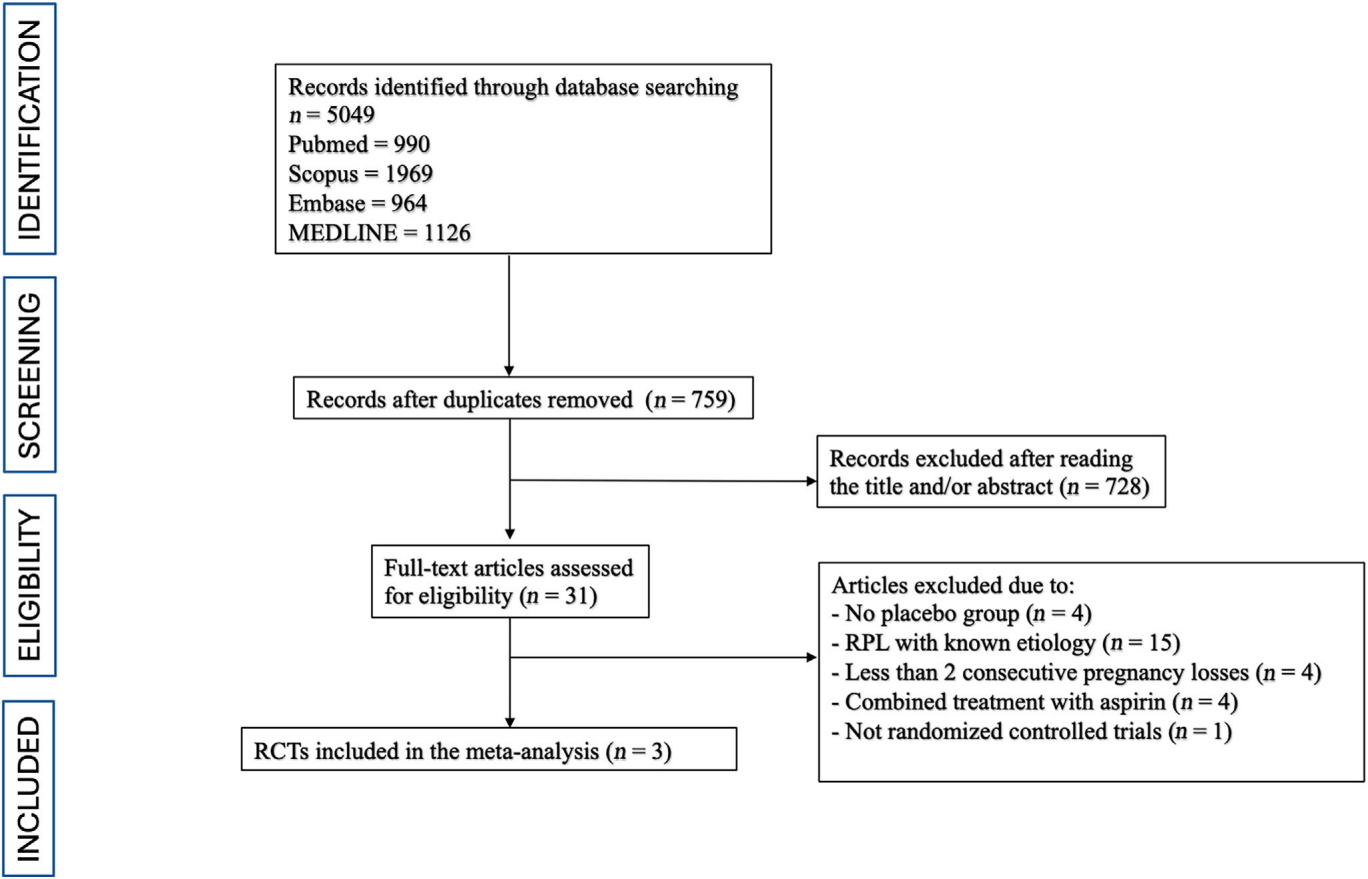
Flowchart of the study selection process. RCTs, randomized controlled trials; RPL, Recurrent pregnancy loss.

The search strategy generated 759 citations after removal of duplicates. Of these, 31 appeared to be relevant and were retrieved for further assessment. In total, 3 RCTs, including a total of 656 subjects, were eligible and were included in the final analysis [15, 16, 17]. A flowchart describing the process for the study selection is shown in Figure 2. Characteristics of the included studies are described in Table 1, while characteristics of the excluded studies [18, 19, 20, 21, 22, 23, 24, 25, 26, 27, 28, 29, 30, 31, 32, 33, 34, 35, 36, 37, 38, 39, 40] are shown in Table S3.

**TABLE 1:** Characteristics of included studies.

Author, year, country	Journal	Study design	Dosage (daily)	Intervention group	Placebo group	Timing	Primary outcome	Secondary outcomes	Findings
Blomqvist et al., 2018, Sweden	*Acta Obstetricia et Gynecologica Scandinavica*	Double blinded RCT	75 mg	200 women with 3 or more uRPL	200 women with 3 or more uRPL	From early confirmation of viable pregnancy until 36 weeks of gestation	LBR	Antenatal vaginal bleeding, placenta previa, PTB, small‐for‐ gestational‐age, PE, pregnancy‐induced hypertension, neonatal morbidity and mortality	LDA does not improve LBR in uRPL: 83.0% (*n* = 166) intervention groups *vs*. 85.5% (*n* = 171) placebo group
Kaandorp et al., 2010, the Netherlands	*The New England Journal of Medicine*	Double blinded RCT	80 mg	99 women with 3 or more uRPL	103 women with 3 or more uRPL	Before conception or at a gestational age of less than 6 weeks until 36 weeks of gestation	LBR	Miscarriage rate, intrauterine fetal death after 20 gw	LDA does not improve LBR in uRPL: 61.6% (*n* = 61) intervention group *vs*. 67% (*n* = 69) placebo group
Tulppala et al., 1997, Finland	*Human Reproduction*	Double blinded RCT	50 mg	27 women with 3 or more uRPL	27 women with 3 or more uRPL	From the positive home pregnancy test, on average 6.0 ± 0.9 and 6.9 ± 0.7 days after the missed period in the groups that received LDA (50 mg) and placebo, respectively, until delivery	LBR	Miscarriage, ectopic pregnancy, PE, IUGR, GDM	LDA does not improve LBR in uRPL: 81.5% (*n* = 22) intervention group *vs*. 81.5% (*n* = 22) placebo group

Abbreviations: GDM, gestational diabetes mellitus; gw, gestational weeks; IUGR, intrauterine growth restriction; LBR, live birth rate; LDA, low‐dose of aspirin; PE, preeclampsia; PTB, preterm birth; RCT, randomized control trial; uRPL, unexplained recurrent pregnancy loss.

In all RCTs included, women with ≥3 uRPL were enrolled [15, 16, 17]. Across the three RCTs included, age distributions were generally comparable, with all participants within reproductive age. The RCT by Blomqvist et al. reported values ranging from a mean of 32.4 ± 4.5 years in the LDA group to 32.1 ± 4.1 years in the placebo group [15], while Kaandorp et al. reported values ranging from a mean of 33 ± 5 years in the LDA group to 34 ± 5 years in the placebo group [16]. On the other hand, RCT by Tulppala et al. provided a pooled mean age of 32.7 ± 0.5 years for both groups combined [17].

All studies were conducted in a single country and subjects were recruited in three different countries (Finland, Sweden, and the Netherlands). In all of the included studies, women with uRPL were screened through standard diagnostic work‐up to exclude known causes such as uterine anomalies, thyroid dysfunction, antiphospholipid antibodies, hormonal or metabolic dysfunctions, autoimmune and thrombophilic disorders, and parental chromosomal abnormalities. Although the diagnostic work‐up differed slightly across studies and groups, the diagnostic approach was aligned with international guidelines for RPL [1, 2]. High agreement between investigators for assessment of study eligibility was detected (Cohen's *κ* statistic = 0.74).

In the RCT by Blomqvist et al., LDA treatment (daily dose of 75 mg) was started after an early confirmation of viable pregnancy until 36 weeks of gestation [15]. In the RCT by Kaandorp et al., a daily administration of 80 mg of LDA started before conception or at a gestational age of less than 6 weeks until 36 weeks of gestation [16]. Finally, in the RCT by Tulppala et al., treatment with 50 mg/day of LDA was started from a positive pregnancy test, on average 6.0 ±  0.9 and 6.9 ±  0.7 days after the missed period in the groups that received LDA (50 mg) and placebo, respectively, until delivery [17].

### 3.2 Live birth rate

**FIGURE 3:**
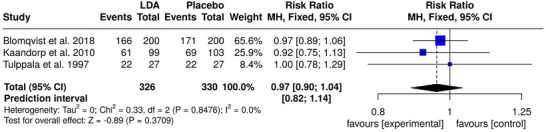
Forest plot of the live birth rate in unexplained recurrent pregnancy loss (uRPL) women daily treated with low dose of aspirin compared to the placebo group. LDA, low‐dose aspirin.

A total of 656 women with uRPL were included in this systematic review and meta‐analysis. Combined data revealed no heterogeneity between studies (*I* ^2 ^= 0%), so a fixed‐effect model was chosen to analyze this outcome parameter. The pooled estimates suggest that the daily administration of LDA was not significantly associated with increased LBR in uRPL women (RR = 0.97, 95% CI, 0.90–1.04, *p* = 0.37, Figure 3).

### 3.3 Secondary pregnancy outcomes

#### 3.3.1 Preeclampsia

**FIGURE 4:**
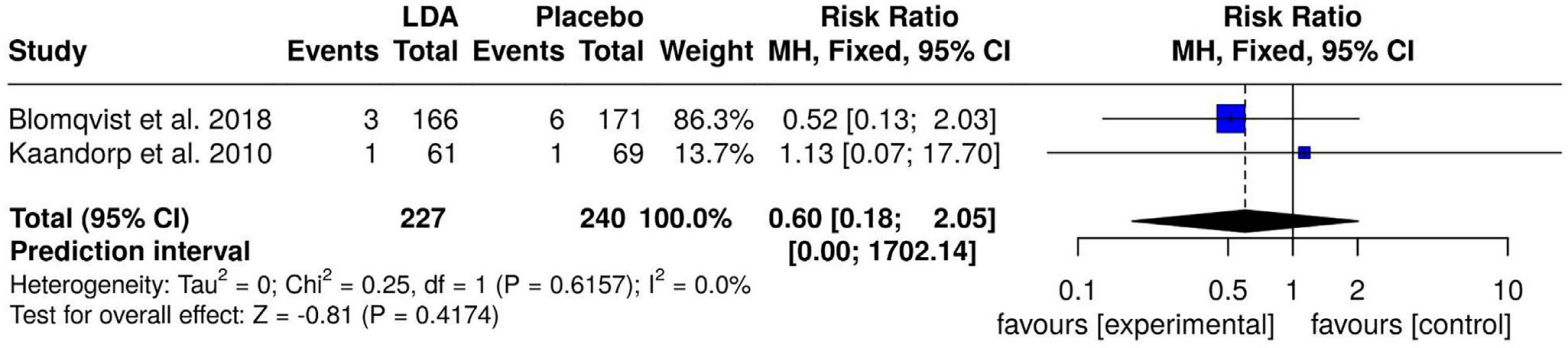
Forest plot of preeclampsia rate in unexplained recurrent pregnancy loss (uRPL) women treated with a low dose of aspirin compared to the placebo group. CI, confidence interval; LDA, low‐dose aspirin.

All included RCTs reported the prevalence of PE among uRPL women who had a live birth, but Tulppala et al. did not specify whether the PE cases were among those with normal anticardiolipin antibodies. For this reason, the meta‐analysis was performed with two RCTs only. The combined data revealed homogeneity between studies (*I* ^2 ^= 0%), so a fixed‐effect model was chosen to analyze this outcome. No significant effect of LDA on the rate of PE was observed among uRPL treated with LDA or placebo (RR = 0.60, 95% CI, 0.18–2.065, *p* = 0.42) (Figure 4).

**FIGURE 5:**
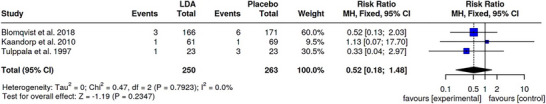
Forest plot of preeclampsia rate in unexplained recurrent pregnancy loss (uRPL) women treated with a low dose of aspirin compared to the placebo group. CI, confidence interval; LDA, low‐dose aspirin.

Given the limited number of studies included, a subsensitivity analysis was performed, including all three RCTs. The combined data revealed homogeneity between studies (*I* ^2 ^= 0%), so a fixed‐effect model was chosen to analyze this outcome. No significant effect of LDA on the rate of PE was observed among uRPL treated with LDA or placebo (RR = 0.52, 95% CI, 0.18–1.48, *p* = 0.23) (Figure 5).

#### 3.3.2 Fetal growth restriction

**FIGURE 6:**
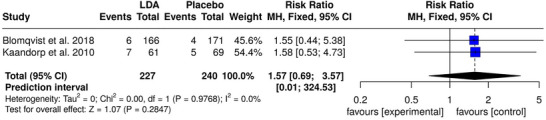
Forest plot of the fetal growth restriction (FGR) rate in unexplained recurrent pregnancy loss (uRPL) women treated with a low dose of aspirin compared to the placebo group. CI, confidence interval; LDA, low‐dose aspirin.

All the three RCTs reported the prevalence of FGR among uRPL women who had a live birth, but Tulppala et al. did not specify whether the FGR cases were among those with normal anticardiolipin antibodies. For this reason, the meta‐analysis was performed with two RCTs only. The combined data revealed homogeneity between studies (*I* ^2 ^= 0%), so a fixed‐effect model was chosen to analyze this outcome. LDA had no effect on the rate of FGR compared to the placebo group (RR = 1.57, 95% CI, 0.69–3.57, *p* = 0.28) (Figure 6).

**FIGURE 7:**
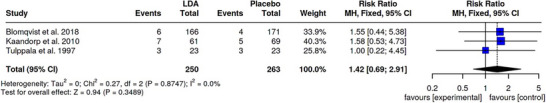
Forest plot of the fetal growth restriction (FGR) rate in unexplained recurrent pregnancy loss (uRPL) women treated with a low dose of aspirin compared to the placebo group. CI, confidence interval; LDA, low‐dose aspirin.

Given the limited number of studies included, a subsensitivity analysis was performed, including all three RCTs. The combined data revealed homogeneity between studies (*I* ^2 ^= 0%), so a fixed‐effect model was chosen to analyze this outcome. No significant effect of LDA on the rate of FGR was observed among uRPL treated with LDA or placebo (RR = 1.42, 95% CI, 0.69–2.91, *p* = 0.35) (Figure 7).

## 4 DISCUSSION

In this systematic review and meta‐analysis, we found that daily administration of LDA (50–80 mg) to uRPL women during pregnancy does not significantly improve the LBR. Furthermore, LDA does not affect FGR nor PE occurrence in uRPL women, carrying on their pregnancies. These results are consistent with other reports on the efficacy of LDA in uRPL [41]. The strength of this study is the strict selection of RCTs testing LDA alone compared to placebo in a uRPL population and the homogeneity among the selected studies. However, the results of this meta‐analysis are substantially limited by the small number of RCTs available (*n* = 3), the small population analyzed (*n* = 656), and the wide confidence interval of the risk ratio for the outcomes FGR and PE.

In general, the evaluation of the efficacy of treatments in the context of RPL is a complex matter, since it is a heterogeneous syndrome with a multifactorial etiology. Several risk factors can be found in this population, isolated or combined in different ways, complicating the clinical individual setting and making it difficult to analyze its pathogenesis as well as clinical efficacy of different treatments. Furthermore, therapies are often tested in combination with other drugs making unfeasible to evaluate the efficacy of any single drug on pregnancy outcome. The selection of uRPL, which represents the 40%–50% of all RPL [2], usually helps to identify a more homogeneous population to test the efficacy of a single therapy. In cases of uRPL, a possible vascular disorder at maternal‐fetal interface leading to placental inadequate development can be supposed, mostly based on the frequent pathological findings of immature villi, hemorrhagic areas, and intervillous thrombosis in unexplained miscarriage [42]. Noteworthily, pathological placental features of miscarriage are similar to those found in PE [8], supporting the hypothesis of a common pathogenesis for miscarriage and PE. Since PE is effectively prevented by the administration of 150 mg/day of aspirin from 11–14 to 36 weeks of gestation [4], LDA might represent, in this setting as well, a valuable therapeutic option for preventing placental insufficiency‐related miscarriage.

Although we could not find any significant effect of aspirin on LBR in uRPL women, in our opinion, the lack of efficacy of aspirin administration in the three trials reported may be also due to the inadequate dosage of aspirin used (50, 75, or 80 mg). Indeed, the effective dose of aspirin used in preventing PE is 150 mg/day [3]. If miscarriage is supposed to be the most severe clinical presentation of placental inadequate development [8], it is unlikely that it might be prevented with the administration of a lower dose than that recommended in PE.

In conclusion, this meta‐analysis did not show that LDA (50–80 mg) is effective in improving LBR and pregnancy outcomes in women with uRPL, but it is limited by the small number of studies included and the low dosage of ASA used. Further RCTs are needed to test the dosage of 150 mg/day of aspirin in uRPL and to better figure out a possible therapeutic role of aspirin in the context of uRPL.

## AUTHOR CONTRIBUTIONS

Chiara Tersigni conceptualized and designed the systematic review and meta‐analysis; Fabio Sannino and Alessandro Petrecca selected the articles, performed data extraction and quality assessment; Marianna Onori and Matteo Figà performed the meta‐analysis. Marianna Onori, Giuliana Beneduce, and Fabio Sannino wrote the original draft of manuscript; Chiara Tersigni and Tullio Ghi revised and edited the manuscript; Chiara Tersigni and Tullio Ghi supervised the overall study design. All authors have read and agreed to the published version of the manuscript.

## CONFLICT OF INTEREST STATEMENT

The authors declare no conflicts of interest.

## Supporting information



Supporting information

## Data Availability

All data generated in this study are included in this published article and in its Supporting Information files.
