# Supplementary material for: Low‐dose aspirin in unexplained recurrent pregnancy loss: A systematic review and meta‐analysis
Source: Pregnancy (Hoboken). 2025 Nov 22;1(6):e70099. doi: 10.1002/pmf2.70099 (PMC13344574; doi:10.1002/pmf2.70099)
Supplement: Supplementary file 1 [file PMF2-1-e70099-s001.docx]

**Supplemental material**

**Table S1. PRISMA checklist**

| **Section and Topic** | **Item #** | **Checklist item** | **Location where item is reported** |
| --- | --- | --- | --- |
| **TITLE** | | |  |
| **Title** | **1** | **Identify the report as a systematic review.** | **1** |
| **ABSTRACT** | | |  |
| **Abstract** | **2** | **See the PRISMA 2020 for Abstracts checklist.** | **2** |
| **INTRODUCTION** | | |  |
| **Rationale** | **3** | **Describe the rationale for the review in the context of existing knowledge.** | **3** |
| **Objectives** | **4** | **Provide an explicit statement of the objective(s) or question(s) the review addresses.** | **3** |
| **METHODS** | | |  |
| **Eligibility criteria** | **5** | **Specify the inclusion and exclusion criteria for the review and how studies were grouped for the syntheses.** | **4** |
| **Information sources** | **6** | **Specify all databases, registers, websites, organisations, reference lists and other sources searched or consulted to identify studies. Specify the date when each source was last searched or consulted.** | **4** |
| **Search strategy** | **7** | **Present the full search strategies for all databases, registers and websites, including any filters and limits used.** | **4** |
| **Selection process** | **8** | **Specify the methods used to decide whether a study met the inclusion criteria of the review, including how many reviewers screened each record and each report retrieved, whether they worked independently, and if applicable, details of automation tools used in the process.** | **4** |
| **Data collection process** | **9** | **Specify the methods used to collect data from reports, including how many reviewers collected data from each report, whether they worked independently, any processes for obtaining or confirming data from study investigators, and if applicable, details of automation tools used in the process.** | **4** |
| **Data items** | **10a** | **List and define all outcomes for which data were sought. Specify whether all results that were compatible with each outcome domain in each study were sought (e.g. for all measures, time points, analyses), and if not, the methods used to decide which results to collect.** | **4** |
|  | **10b** | **List and define all other variables for which data were sought (e.g. participant and intervention characteristics, funding sources). Describe any assumptions made about any missing or unclear information.** | **4** |
| **Study risk of bias assessment** | **11** | **Specify the methods used to assess risk of bias in the included studies, including details of the tool(s) used, how many reviewers assessed each study and whether they worked independently, and if applicable, details of automation tools used in the process.** | **4-5** |
| **Effect measures** | **12** | **Specify for each outcome the effect measure(s) (e.g. risk ratio, mean difference) used in the synthesis or presentation of results.** | **5** |
| **Synthesis methods** | **13a** | **Describe the processes used to decide which studies were eligible for each synthesis (e.g. tabulating the study intervention characteristics and comparing against the planned groups for each synthesis (item #5)).** | **5** |
|  | **13b** | **Describe any methods required to prepare the data for presentation or synthesis, such as handling of missing summary statistics, or data conversions.** | **5** |
|  | **13c** | **Describe any methods used to tabulate or visually display results of individual studies and syntheses.** | **4-5** |
|  | **13d** | **Describe any methods used to synthesize results and provide a rationale for the choice(s). If meta-analysis was performed, describe the model(s), method(s) to identify the presence and extent of statistical heterogeneity, and software package(s) used.** | **4-5** |
|  | **13e** | **Describe any methods used to explore possible causes of heterogeneity among study results (e.g. subgroup analysis, meta-regression).** | **4-5** |
|  | **13f** | **Describe any sensitivity analyses conducted to assess robustness of the synthesized results.** | **4-5** |
| **Reporting bias assessment** | **14** | **Describe any methods used to assess risk of bias due to missing results in a synthesis (arising from reporting biases).** | **4-5** |
| **Certainty assessment** | **15** | **Describe any methods used to assess certainty (or confidence) in the body of evidence for an outcome.** | **4-5** |
| **RESULTS** | | |  |
| **Study selection** | **16a** | **Describe the results of the search and selection process, from the number of records identified in the search to the number of studies included in the review, ideally using a flow diagram.** | **6** |
|  | **16b** | **Cite studies that might appear to meet the inclusion criteria, but which were excluded, and explain why they were excluded.** | **6-7** |
| **Study characteristics** | **17** | **Cite each included study and present its characteristics.** | **8** |
| **Risk of bias in studies** | **18** | **Present assessments of risk of bias for each included study.** | **Supplem.** |
| **Results of individual studies** | **19** | **For all outcomes, present, for each study: (a) summary statistics for each group (where appropriate) and (b) an effect estimate and its precision (e.g. confidence/credible interval), ideally using structured tables or plots.** | **8** |
| **Results of syntheses** | **20a** | **For each synthesis, briefly summarise the characteristics and risk of bias among contributing studies.** | **8** |
|  | **20b** | **Present results of all statistical syntheses conducted. If meta-analysis was done, present for each the summary estimate and its precision (e.g. confidence/credible interval) and measures of statistical heterogeneity. If comparing groups, describe the direction of the effect.** | **9-10** |
|  | **20c** | **Present results of all investigations of possible causes of heterogeneity among study results.** | **9-10** |
|  | **20d** | **Present results of all sensitivity analyses conducted to assess the robustness of the synthesized results.** | **9-10** |
| **Reporting biases** | **21** | **Present assessments of risk of bias due to missing results (arising from reporting biases) for each synthesis assessed.** | **NA** |
| **Certainty of evidence** | **1** | **Present assessments of certainty (or confidence) in the body of evidence for each outcome assessed.** | **9-10** |
| **DISCUSSION** | | |  |
| **Discussion** | **23a** | **Provide a general interpretation of the results in the context of other evidence.** | **10-11** |
|  | **23b** | **Discuss any limitations of the evidence included in the review.** | **10-11** |
|  | **23c** | **Discuss any limitations of the review processes used.** | **10-11** |
|  | **23d** | **Discuss implications of the results for practice, policy, and future research.** | **10-11** |
| **OTHER INFORMATION** | | |  |
| **Registration and protocol** | **24a** | **Provide registration information for the review, including register name and registration number, or state that the review was not registered.** | **5** |
|  | **24b** | **Indicate where the review protocol can be accessed, or state that a protocol was not prepared.** | **NA** |
|  | **24c** | **Describe and explain any amendments to information provided at registration or in the protocol.** | **NA** |
| **Support** | **25** | **Describe sources of financial or non-financial support for the review, and the role of the funders or sponsors in the review.** | **11** |
| **Competing interests** | **26** | **Declare any competing interests of review authors.** | **11** |
| **Availability of data, code and other materials** | **27** | **Report which of the following are publicly available and where they can be found: template data collection forms; data extracted from included studies; data used for all analyses; analytic code; any other materials used in the review.** | **NA** |

**Search strategy**

PubMed, PROSPERO, Scopus, Embase, MEDLINE, Google Scholar and Cochrane Library were systematically searched without language restriction to identify potentially eligible studies. The search time frame was from inception up to April 2025. To exclude studies that did not meet the eligibility criteria, two investigators (FS and AP) independently assessed titles and abstracts of all studies, searched the literature and reported effect size (risk ratio). Conflicts in study selection were resolved by consulting a third reviewer (MO).

**Search string**

((Aspirin) OR (cardioaspirin) OR (ASA) OR (acetylsalicylic acid) OR (low dose aspirin) AND (recurrent pregnancy loss) OR (recurrent miscarriage) OR (unexplained recurrent pregnancy loss) OR (idiopathic recurrent pregnancy loss)).

**Table S2.** Methodological quality (JBI) of RCTs included in the meta-analysis.

| **Study** | **Q1** | **Q2** | **Q3** | **Q4** | **Q5** | **Q6** | **Q7** | **Q8** | **Q9** | **Q10** | **Q11** | **Q12** | **Q13** |
| --- | --- | --- | --- | --- | --- | --- | --- | --- | --- | --- | --- | --- | --- |
| Blomqvist et al., 2018 | Y | Y | Y | Y | Y | Y | Y | Y | Y | Y | Y | Y | Y |
| Kaandorp et al., 2010 | Y | Y | Y | Y | Y | Y | Y | Y | Y | Y | Y | Y | Y |
| Tulppala et al., 1997 | Y | Y | Y | Y | Y | Y | Y | Y | Y | Y | Y | Y | Y |
| % | 100 | 100 | 100 | 100 | 100 | 100 | 100 | 100 | 100 | 100 | 100 | 100 | 100 |

N=No; U = Unclear; Y = Yes; N/A= not applicable.

**Table S3.** Excluded studies.

| **Reference** | **Author, year & country** | **Journal** | **Study design** | **Reasons of exclusion** |
| --- | --- | --- | --- | --- |
| 18 | Schisterman et al., 2014 | Lancet | RCT | Less than 2 consecutive pregnancy losses |
| 19 | Mumford et al., 2016 | Human Reproduction | RCT | Less than 2 consecutive pregnancy losses |
| 20 | Dolitzky et al.; 2006 | Fertility and Sterility | RCT | No placebo group (40 mg of enoxaparin *versus* 100 mg of aspirin) |
| 21 | Visser et al., 2011 | Thrombosis and Haemostasis | RCT | No placebo group (40 mg of enoxaparin and placebo/ 40 mg of enoxaparin and 100 mg aspirin/100 mg aspirin alone) |
| 22 | Laskin et al., 2009 | Journal of Rheumatology | RCT | Population selection bias (RPL women with antiphospholipid antibody/thrombophilia/antinuclear antibody) |
| 23 | Clark et al., 2010 | Blood | RCT | Combined treatment (40 mg of enoxaparin and 75 mg of aspirin) |
| 24 | Giancotti et al., 2012 | The Journal of Maternal-Fetal& Neonatal Medicine | RCT | Population selection bias (RPL women with and without thrombophilia) |
| 25 | Karadağ et al., 2019 | The Journal of Maternal-Fetal& Neonatal Medicine | RCT | Population selection bias (RPL women with factor V Leiden mutation) and  no placebo group (100 mg aspirin alone/40 mg enoxaparin and 100 mg aspirin/ 40 mg enoxaparin alone) |
| 26 | Ogasawara et al., 2001 | Fertility and Sterility | CT | Population selection bias (RPL women with decreased coagulation factor XII) |
| 27 | Reznikoff-Etievant et al., 1999 | Human Reproduction | CT | No placebo group (20 mg of prednisone and 100 mg of aspirin/ 100 mg of aspirin alone) |
| 28 | Laskin et al., 1997 | NEJM | RCT | RPL women with positive results of at least one of the following: activated partial-thromboplastin time test or tests for antinuclear antibodies, anti-DNA antibodies (single- or double-stranded), antilymphocyte IgM, anticardiolipin IgG, or the lupus anticoagulant |
| 29 | Ou et al., 2017 | Zhonghua Yi Xue Za Zhi | CT | Combined treatment (aspirin, prednisone and Elevit) *versus* placebo |
| 30 | Kutteh et al., 1996 | Am J Obstet Gynecol | CT | RPL with antiphospholipid antibody syndrome |
| 31 | Alalaf et al., 2012 | Arch Gynecol Obstet | RCT | RPL with antiphospholipid antibody syndrome |
| 32 | Carta et al., 2005 | Clin Exp Obstet Gynecol | CT | RPL with antiphospholipid antibody syndrome |
| 33 | Schisterman et al., 2013 | Paediatr Perinat Epidemiol | RCT | Less than 2 consecutive pregnancy losses |
| 34 | Farquharson et al., 2002 | Obstet Gynecol | CT | RPL with persistently positive tests for lupus anticoagulant and/or anticardiolipin immunoglobulin G and M antibody |
| 35 | A/Magid et al., 2007 | Afr J Reprod Health | CT | RPL with antiphospholipid antibody syndrome |
| 36 | Pattison et al., 2000 | Am J Obstet Gynecol | RCT | RPL with antiphospholipid antibody syndrome |
| 37 | Elmahash et al., 2014 | BMC Res Notes | CT | No placebo group (75 mg of LDA alone or a combination of 75 mg of LDA and 0.4 mL of LMWH) |
| 38 | Rai et al., 1997 | BMJ | RCT | RPL with antiphospholipid antibody syndrome |
| 39 | Goel et al., 2006 | Med Sci Monit | RCT | No placebo group (80 mg of LDA alone or a combination of 80 mg LDA and 5000 IU of unfractionated heparin subcutaneously) |
| 40 | Fawzy et al., 2008 | Arch Gynecol Obstet | RCT | Combined treatment (enoxaparin alone, combination treatment consisting of prednisone, aspirin, and progesterone or placebo) |
| 41 | Rai et al., 2000 | Human Reproduction | CT | Not randomized controlled trial |

*RCT: randomized clinical trial; CT: clinical trial; RPL: recurrent pregnancy loss; LDA: low dose aspirin; LMWH: low-molecular-weight-heparin; mg: milligrams; mL: milliliter; IU: International Unit.*
